# Reliability of 95% confidence interval revealed by expected quality-of-life scores: an example of nasopharyngeal carcinoma patients after radiotherapy using EORTC QLQ-C 30

**DOI:** 10.1186/1477-7525-8-68

**Published:** 2010-07-13

**Authors:** Tsair-Wei Chien, Shun-Jin Lin, Wen-Chung Wang, Henry WC Leung, Wen-Pin Lai, Agnes LF Chan

**Affiliations:** 1 Department of Pharmacy, Chi-Mei Medical Center, Tainan, Taiwan; 2 Department of Hospital and Health Care Administration, Chia-Nan University of Pharmacy and Science, Tainan, Taiwan; 3 School of Pharmacy, Kaohsiung Medical University, Kaohsiung, Taiwan; 4 Assessment Research Center, The Hong Kong Institute of Education, Tai Po, Hong Kong, China; 5 Department of Radiation Oncology, Taipei Medical University, Shuang Ho Hospital, Taipei, Taiwan; 6 Department of Emergency, Chi-Mei Medical Center, Tainan, Taiwan

## Abstract

**Background:**

Many researchers use observed questionnaire scores to evaluate score reliability and to make conclusions and inferences regarding quality-of-life outcomes. The amount of false alarms from medical diagnoses that would be avoided if observed scores were substituted with expected scores is interesting, and understanding these differences is important for the care of cancer patients. Using expected scores to estimate the reliability of 95% confidence intervals (CIs) is rarely reported in published papers. We investigated the reliability of patient responses to a quality-of-life questionnaire and made recommendations for future studies of the quality of life of patients.

**Methods:**

A total of 115 patients completed the EORTC core questionnaire QLQ-C30 (version 3) after radiotherapy. The observed response scores, assumed to be one-dimensional, were summed and transformed into expected scores using the Rasch rating scale model with WINSTEPS software. A series of simulations was performed using a unified bootstrap procedure after manipulating scenarios with different questionnaire lengths and patient numbers to estimate the reliability at 95% confidence intervals. Skewness analyses of the 95% CIs were compared to detect different effects between groups according to the two data sets of observed and expected response scores.

**Results:**

We found that (1) it is necessary to report CIs for reliability and skewness coefficients in papers; (2) data derived from expected response scores are preferable to making inferences; and (3) visual representations displaying the 95% CIs of skewness values applied to item-by-item analyses can provide a useful interpretation of quality-of-life outcomes.

**Conclusion:**

Reliability coefficients can be reported with 95% CIs by statistical software to evaluate the internal consistency of respondent scores on questionnaire items. The SPSS syntax procedures for estimating the reliability of the 95% CI, expected score generation and visual skewness analyses are demonstrated in this study. We recommend that effect sizes such as a 95% CI be reported along with *p *values reporting significant differences in quality-of-life studies.

## Background

Cronbach's *α *coefficient (hereinafter referred to as Alpha [1]) is widely used as an index of scoring reliability and is often reported in social and behavioral studies [2,3]. However, very few authors report the confidence intervals (CIs) of Alpha in their papers, although this has been suggested by many researchers [4-7].

### Conception of research questions

The American Psychological Association Task Force on Statistical Inference suggests, "ways provide some effect-size estimate when reporting a *p *value" [4]. The task force also noted that "interpreting the size of observed effects requires an assessment of the reliability of the scores" because score unreliability weakens effect size [5]. Confidence intervals around Alpha are advocated for both absolute and relative decisions made by researchers [6,7]. Alexander et al., in their critical review of research methods regarding quality improvement research, also stated that 74.1% of the studied articles discussed effect size [8]. These reports underscore the need for research on the disclosure of Alpha and 95% CIs in quality-of-life studies.

Most research on quality of life uses observed scores obtained from surveys and questionnaires to make conclusions and inferences regarding quality-of-life outcomes. False responses on patient questionnaires can be caused by aberrant answers, guessing, inattentiveness (which may result from fatigue), misunderstanding or response misfit, according to item response theory [9,10]. These issues can result in false alarms (i.e., false positive and false negative errors) coming from medical diagnoses and leading to make vain efforts in actions taking. It is of interest to discuss the possibility of replacing observed response scores with expected ones to make decisions and inferences by applying probability theory to studies of quality-of-life outcomes.

### Objectives

This article is organized as follows. First, a set of EORTC QLQ-C30 component items developed to assess the quality of life of cancer patients [11] was given to 115 patients who were diagnosed with nasopharyngeal carcinoma. Second, we present a brief introduction of the Rasch [12] model, which we performed to determine expected response scores by the probability function of item difficulty and person ability. Third, Alphas for 95% CIs were generated by a SPSS syntax procedures for reliability 95% CI estimation referring to both observed and expected response scores on EORTC QLQ-C30 by manipulating different person numbers. Fourth, graphical representations of skewness analyses with 95% CIs were applied to justify differences between cancer stages across items, by which the misfitted items on the EORTC QLQ-C30 were examined in further detail with both observed and expected response scores.

## Methods

### Research data resource

A total of 115 patients newly diagnosed with nasopharyngeal carcinoma were recruited to answer the first 28 questions on the Taiwanese version of the EORTC QLQ-C30 questionnaire, which requires answers on a scale of 1 to 4 (1 = Not at all, 2 = A little, 3 = Quite a bit, and 4 = Very much). The two composite questions requiring answers on a 1-7 scale were excluded from the study ("How would you rate your overall health during the past week?" and "How would you rate your overall quality of life during the past week?"). All examinees were treated in one of three Taiwanese medical centers and had a follow-up evaluation a minimum of three months later without complete remission. Accordingly, the 115-person × 28-item observed-response matrix was created. This study was approved by the institutional review boards of the hospitals.

### Data analysis using the Rasch model

We assumed that the 28-item EORTC QLQ-C30 fits Rasch model's specification and forms a unidimensional construct. If any item could not fit the Rasch model expectation, patients were deemed to have exhibited unexpected behaviors, such as aberrant responses, guessing or inattentiveness, which may have led to responses outside of the model's expectations (as we assumed all 28 items constructed a one-dimensional latent trait).

When the data fit the model's expectations, the infit and outfit mean square error (MNSQ) statistics had an expected value of unity on the items. The values of the MNSQ statistics show the amount of distortion of the measurement system. Values less than unity (or over-fit) showed that the items were too predictable (i.e., there was redundancy). Values greater than unity (or under-fit) indicated unpredictability (i.e., noise). The MNSQ statistics were chi-square statistics divided by their degrees of freedom. Items with infit MNSQ beyond the range of 0.5-1.5 were usually regarded as misfitting or poor-fitting [13,14].

### Excel module for data simulation

For our approach to succeed, an Excel-VBA(visual basic for application) module according to the formulas of Cronbach's *α *(see Formula 1) and skewness (see Formula 2) was programmed.

(1) $$\alpha =\frac{N}{N-1}(1-\frac{\sum_{i=1}^{N}\sigma_{Yi_{i}}^{2}}{\sigma_{x}^{2}}),$$

Cronbach's *α *is defined in Formula 1, where *N *is the number of items, $\sigma_{x}^{2}$ is the variance of the observed total test scores for the sample of examinees and $\sigma_{Yi}^{2}$ is the variance of item i for the sample of examinees.

Formula 2 shows the equation for skewness:

(2) $$Skewness=\frac{n}{\left(n-1)(n-2\right)}\sum {\left(\frac{x_{i}-\bar{x}}{s}\right)}^{3},$$

where *n *is the number of responses, *x*_*i *_is the value of individual responses and $\bar{x}$ and s denote the mean and standard deviation of the total responses, respectively.

(3) $$p_{nix}=\frac{\exp(\sum_{j=0}^{x}(\theta_{n}\text{-}\delta_{\text{ij}})}{\sum_{k=0}^{mi}\exp(\sum_{j=0}^{k}(\theta_{n}\text{-}\delta_{\text{ij}})},$$

In Formula 3, based on the Rasch rating scale model [15] shown in Additional file 1, the probability of a person *n *in a specific category *j *of item *i *is yielded by person ability *θ *and item difficulty *δ *on an *m*-point scaled questionnaire.

The expected response scores would be generated by Rasch model's probability function of item difficulties and person measures (see Additional file 1). We anticipate that the person expected scores could produce item MNSQ statistics between 0.5 and 1.5 when item difficulties are anchored according to the original 115 (person) × 28 (item) observed response data set.

The author-programmed A Excel-VBA module for computing test Cronbach's *α*, item/person skewness coefficient and person expected response score generation is shown (in Additional file 2) according to the item difficulties (Table 1) yielded by WINSTEPS [16] using the Rasch rating scale model [15] from the original 115 (person) × 28 (item) observed matrix.

**Table 1:** The EORTC QLQ-C30 questionnaire, sorted in descending order of difficulty (logit units) according to the patient responses in this study (questions 29 and 30, which were not reported, are excluded)

		Difficulty	Measure	Observed	Expected
**No**.	Items	Logit*	SE	IN.MSQ	IN.MSQ
5	Do you need help with eating, dressing, washing yourself or using the toilet?	2.4	0.31	***1.60***	1.21
3	Do you have any trouble taking a short walk outside of the house?	1.45	0.23	1.04	0.85
15	Have you vomited?	1.15	0.21	1.61	1.2
17	Have you had diarrhea?	0.97	0.21	1.17	0.93
8	Were you short of breath?	0.73	0.2	1.2	0.91
6	Were you limited in doing either your work or other daily activities?	0.69	0.19	0.95	1.03
16	Have you been constipated?	0.66	0.19	1.19	0.87
14	Have you felt nauseated?	0.58	0.19	1.41	1.09
7	Were you limited in pursuing your hobbies or other leisure time activities?	0.31	0.18	1.05	0.77
19	Did pain interfere with your daily activities?	0.28	0.18	1.05	0.91
20	Difficulty in concentrating on things, like reading papers or watching TV?	0.25	0.18	1.01	0.91
23	Did you feel irritable?	-0.11	0.17	0.88	1.1
13	Have you lacked appetite?	-0.14	0.17	0.88	1.19
4	Do you need to stay in bed or a chair during the day?	-0.17	0.17	1.16	1.1
1	Trouble doing strenuous activities, like carrying a heavy bag or a suitcase?	-0.19	0.17	0.89	1.03
9	Have you had pain?	-0.22	0.17	1.21	0.96
21	Did you feel tense?	-0.25	0.17	0.87	0.84
25	Have you had difficulty remembering things?	-0.33	0.17	0.75	1.08
2	Do you have any trouble taking a long walk?	-0.41	0.16	1.09	1.09
22	Did you worry?	-0.68	0.16	0.71	1.17
11	Have you had trouble sleeping?	-0.72	0.16	1.31	0.84
26	Physical condition or medical treatment interfered with your family life?	-0.75	0.16	1.41	0.98
24	Did you feel depressed?	-0.77	0.16	0.64	1
27	Physical condition or medical treatment interfered with your social activities?	-0.82	0.16	1.14	0.81
10	Did you need to rest?	-0.84	0.15	0.49	1.13
12	Have you felt weak?	-0.99	0.15	0.41	1.26
28	Physical condition or medical treatment caused you financial difficulties?	-1.02	0.15	***1.74***	1.09
18	Were you tired?	-1.08	0.15	0.49	0.88

*Step threshold difficulties are -1.88, 0.73 and 1.15 under the Rasch rating scale model.

### Procedures for data simulation

#### 1. Alpha 95% CIs by both observed and expected scores

The present study built on the data that were collected from the 115 patients answering the aforementioned 28 items on the EORTC QLQ-C30. As mentioned above, we investigated 95% CIs from the perspective of Alpha by examining different person numbers, such as 115, double(230) and trible(445) with a SPSS syntax procedures (see Additional file 3) referring to both observed and expected scores on EORTC QLQ-C30 to examine any difference between scenarios.

#### 2. Generating expected response data sets by simulation

The random response, for instance with a dichotomous scale, less than the probability $P_{ni}=\frac{\exp(\theta_{n}-\delta_{i})}{1+\exp(\theta_{n}-\delta_{i})}$, an item response of 1 would be assigned for that examinee on the item. On the other hand, had the random number been greater than the response probability, an item response of 0 would be recorded. This is a standard item response generation method as used by previously published papers [17-19]. We can easily extend it to a polytomy-like EORTC QLQ-C30 to generate the expected item response data. The Rasch rating scale model [15] probability generation program is shown in Additional file 1 and was applied to yield a data set of expected response scores constructed by the 1-4 scaling scheme.

#### 3. Item-by-item skewness analysis by a bootstrapping procedure

To compare the quality of life on the 28-item EORTC QLQ-C30 questionnaire, we applied item-by-item comparisons [20,21] of the patient attitudes according to cancer stage. For this purpose, skewness analyses were performed by a bootstrapping method [22-24], sampling with replacement of patient responses at each item, each time selecting 500 responses with 100 replications in ascending order to filter out the median skewness and its 95% CI.

The correlation coefficients between item difficulties (in logit (log odds) units calibrated by WINSTEPS), observed and expected response summation scores across items and the skewness yielded by both observed and expected responses on items are reported. We will examine whether the skewness of expected responses can earn a higher correlation to other counterparts than the skewness of observed scores. A graphical representation of the comparison of patient attitudes toward quality of life across the entirety of the questionnaire will be presented.

### Statistical analyses

Statistical analyses and plotting were performed by SPSS software for Windows (Version 15, SPSS, Chicago, IL).

## Results

### Item difficulties of EORTC QLQ-C30

The most difficult item was question 5 (Do you need help with eating, dressing, washing yourself or using the toilet?), as shown in Table 1. The easiest question was 18 (Were you tired?). Expected response scores, as anticipated, presented an acceptable infit MNSQ more often than observed scores, which displayed two items outside of the range 0.5-1.5. From the observed score perspective, question 5 had an infit MNSQ of 1.6, and question 28 had an infit MNSQ of 1.74, indicating some aberrant responses on those two questions that negatively affected the inferences and conclusions that were made.

### Alpha values for different person numbers

In Table 2 we can see that expected scores earn higher Alpha and its 95% CIs across person numbers than the observed responses. The more person number can produce the narrower 95% CI band.

**Table 2:** Comparisons of test reliability between observed and expected scores by numbers of person.

Response Types	Numbers of Person	Test Reliability	95% Confidence Interval	
			Lower Bound	Upper Bound
Observed scores	115	0.909	0.883	0.931
	230	0.909	0.891	0.925
	345	0.909	0.894	0.922

Expected scores	115	0.920	0.898	0.94
	230	0.920	0.905	0.934
	345	0.920	0.908	0.932

### Skewness analysis with 95% CI on misfitting items

**Figure 1:**
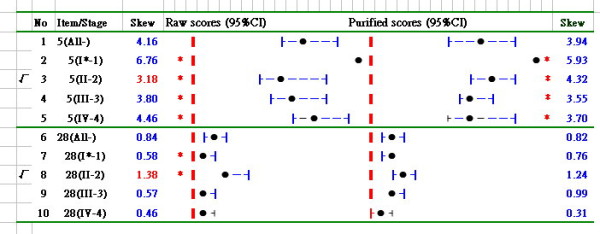
**Skewness analyses of questions 5 and 28 of the EORTC QLQ-C30**. *, statistically significant difference in the cancer stage groups by the band of 95% CI not overlaied (the dot denotes point estimation of skewness coefficient; the border line in red represents the value of skewness coefficient equal to zero).

As shown in Table 1, questions 5 and 28 exhibited greater infit MNSQ errors, indicating some non-randomized, or, say, unexpected aberrant responses on these two items. The skewness and 95% CI of these two misfitted items, calculated from the two data sets of observed and expected scores, are shown on both sides of Figure 1. Significant differences were seen in stage I by the 95% CI far from others of stage II, III and VI on item 5, for which skewness only in stage II displayed a lower value (3.18) by observed scores than by expected scores, (4.32). On item 28, observed raw scores showed a significant difference between stages I and II, but the expected scores did not. When using Rasch measures to generate expected scores for prediction, slightly better results might be obtained when the measure estimates are based on adjusted data. A productive adjustment makes observations in extreme categories slightly less extreme[16].

### Overall skewness analysis with 95% CI across items

**Figure 2:**
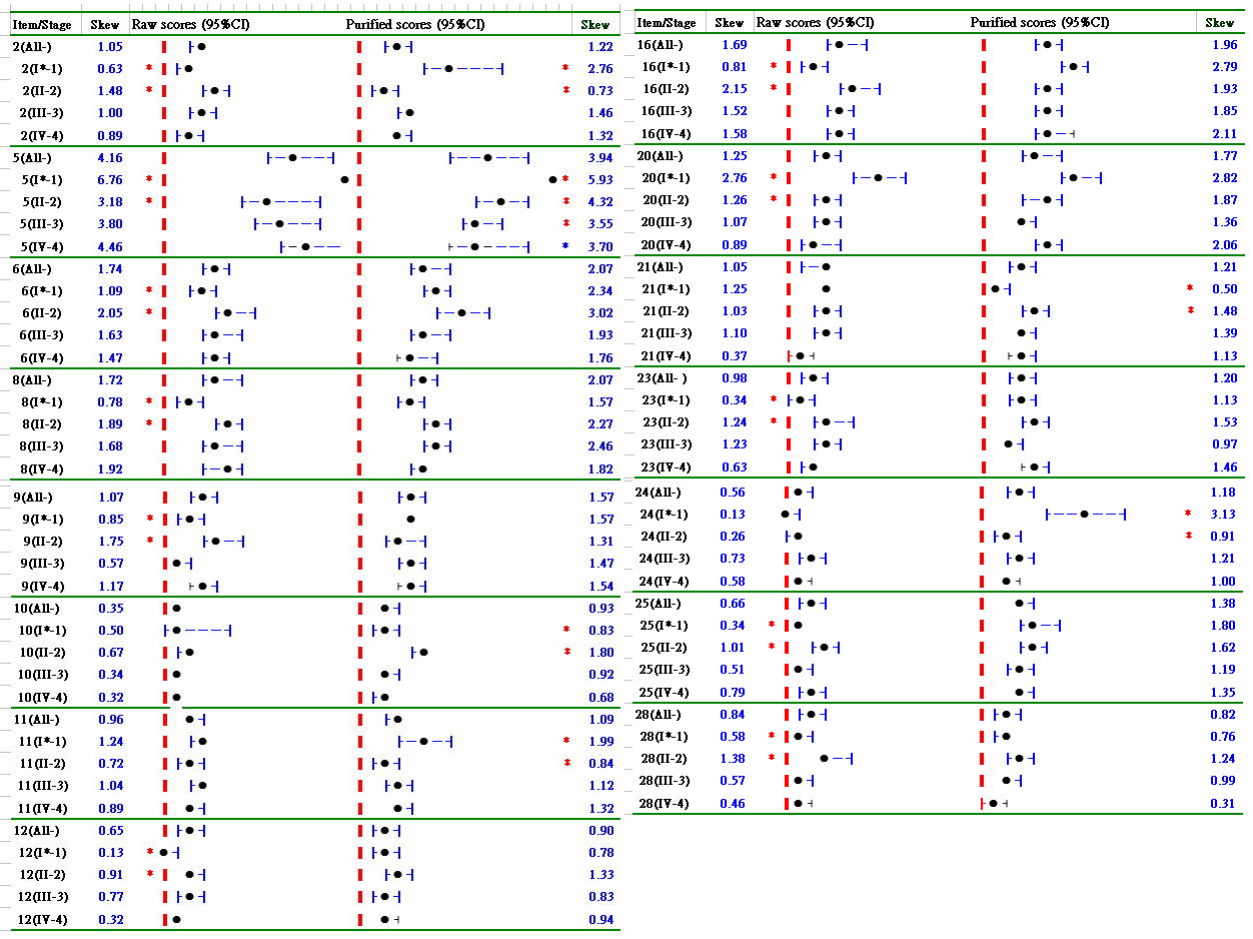
**Skewness analyses for EORTC QLQ-C30 by an item-by-item approach of cancer stages**. *, statistically significant difference in the cancer stage groups by the band of 95% CI not overlaied (the dot denotes point estimation of skewness coefficient; the border line in red represents the value of skewness coefficient equal to zero).

In Figure 2 we can see that there were 6 items on stages showing significant differences in quality-of-life attitude by the expected response scores, but 11 items on stages gave significant differences by the observed response scores, indicating different inferences would have been made if we had chosen different data sources (i.e., observed and expected scores in Figure 2).

As shown in Table 3, the skewness for expected response scores had a higher correlation coefficient (≥ 0.90) than the skewness with observed scores (some ≤ 0.90), as compared to other counterparts, such as Rasch transformed item difficulties (which were derived from original observed response scores), observed and expected summation response scores. This indicates that the expected scores would be more meaningful than the observed scores for making inferences.

**Table 3:** Relationship between items among difficulty (logit), skewness, observed scores and expected scores (item number = 28).

	Difficulty	**Skew_ Exp**.	Observed	Expected	**Skew_ Obs**.
Difficulty	**0.72**	0.55	-21.72	-20.93	0.64
Skew_Exp.	0.95	**0.46**	-16.03	-16.07	0.51
Observed	-0.98	-0.90	**684.07**	656.29	-18.28
Expected	-0.97	-0.93	0.98	**650.99**	-17.58
Skew_Obs.	0.95	0.94	-0.88	-0.87	**0.63**

1. Covariance in upper triangle, correlation coefficient in lower triangle; variance along the diagonal line.

2. Skew_ Exp: skewness with expected scores; Skew_ Obs: skewness with observed scores

## Discussion

The reliability of a test in a given population is defined as the ratio of true score variance to observed score variance [25]. In empirical research, true scores cannot be directly determined. One of the most frequently used methods of estimating internal consistency reliability is Cronbach's coefficient *α*, as shown in Formula 1, which is frequently cited (at least 5,590 times, including approximately 325 social science citations, per year) [2]. Our data indicate that CIs for Cronbach's *α *should be more widely calculated and reported for published data. The SPSS syntax procedure of intervals for reliability coefficients is included in Additional file 3, although it can be estimated in various ways [7]. We hope that in the future, authors will report Cronbach's *α *routinely and note what estimation methods they use. This would help clarify their score reliabilities and how these are affected by sampling error variance.

## Findings

We utilized the Rasch model, using the term "ability" for a latent trait that underlies the questionnaire responses of the participants, to determine the probability of each examinee answering a given question accurately [26]. The expected response scores could then be derived from the original observed data matrix, which we used to obtain a more accurate Alpha and its intervals. Furthermore, the graphical representation of item-by-item skewness analyses using expected response scores was plotted to make more appropriate inferences and conclusions of difference detection among groups of interest, particularly on misfit responses.

## Strengths of the study

Most data analysis of quality-of-life questionnaires is based on classic test theory (CTT). In recent years, CTT has been gradually replaced by item response theory (IRT) [10,14,27,28]. Whereas CTT concerns the accuracy of observed scores, IRT concerns the accuracy of ability scores [29]. In this study, we applied IRT-based Rasch analysis to generate expected response scores and confidence intervals, approaching a more accurate understanding of our patient population and enabling proper treatment decisions.

From Figure 1 and 2 we can see that the 95%CIs for an one-samnple case far from the skewness value of zero is deemed that a statistically significant difference in an attitude toward positive (with a negatively skewed distribution if higher scores represent positive) or toward negative (with a positively skewed distribution if lower scores represent negative) is found (*p *< .05). However, for the two-sample case it should be cautious about interpreting the graphical reports when examining the attitude difference between groups by non-overlapping 95% confidence intervals (CIs). According to the criterion of 95% CIs derived from two independent samples(e.g., A and B), there is a distance beyond 2*SE_A + 2*SE_B between those two means with more than 95% confidence to regard as a significant difference emerged between two groups. That is said that (1) bootstrapping approach is especially suited to data following a non-normal distribution; (2) the strict criterion of 95% CIs derived from two independent samples shows that MB - MA > 2 × SE_A + 2 × SE_B > 2 × sqrt(SE_A^2 + SE_B^2) which holds a high confidence to regard as a significant difference emerged in two groups by checking non-overlapping 95% confidence intervals (CIs).

## Limitations of the study

We simulated data from 115 patients newly diagnosed with nasopharyngeal carcinoma who responded to the 28 questions on the Taiwanese version of the EORTC QLQ-C30 questionnaire that requires answers on a scale of 1 to 4. Some bias might have occurred as a result of the Taiwanese translation of the questionnaire. If so, the item-calibrated difficulties, including step threshold difficulties, would be different among patients from different countries, which may lead to a variety of expected response scores by the Rasch model. The Alpha and 95% CIs shown in Table 2, according to sample size, would also be different in relation to different types of response outliers [9], different test forms, different numbers of questions, misspecifications and confounding independent variables in a single reliability generalization analysis [28].

The Cronbach's alpha has been frequently reported in CTT for a long time though Rasch person separation reliability was reported better than Cronbach's alpha as a test indication in recent years [28]. We do not decline the use of WINSTEPS command by SIFILE = data file name to simulate a data file equivalent to the raw data to obtain Rasch person separation reliability 95% CIs when demonstrating the use of Cronbach's alpha 95% CIs in Additional file 3.

In this study we assumed that the 28-item EORTC QLQ-C30 fits Rasch model's specification and forms a unidimensional construct. However, to our knowledge till now, it has not been either previously tested or reported using a Rasch analysis rather than sample and item dependent classical test models. For the reason of space limitation for this manuscript, we have not explored the dimensionality and invariance of the EORTC QLQ-C30 like the General Health Questionnaire (GHQ)-12 did [30] in detail through the Rasch model in this paper. A brief report regarding dimension and invariance of items has been enclosed in Additional file 4.

## Applications

There are concerns that the expected response scores derived from observed raw scores may also yield unreliable results. The frequent counts of total items (similar to the item difficulties in this study) endorsed by examinees would not be overly distorted if we assume that the errors made by them were randomized across the questions. The specific misfit items observed by a particular group (e.g., stage II cancer patients on item 28 in Figure 2) could be well adjusted by expected response scores generated by the Rasch model's probability theory. In addition, the shortcoming of missing data handled in CTT could be overcome through the derived measures estimated by Rasch modeling.

The SPSS syntax procedures in Additional file 3, which has been revised referring to the previously published article [7], can help authors easily report Alpha intervals in studies involving quality-of-life investigation and surveillance. Furthermore, the skewness coefficient and its 95% CI, referring to expected response scores, can be used to verify whether the assessed attitude is neutral (approaching a normal distribution), in absolute agreement (with a negatively skewed distribution if higher scores represent agreement) or in disagreement (with a positively skewed distribution if lower scores represent disagreement). Use of this coefficient merits further study in other kinds of quality-of-life surveys. One major obstacle is that users may need some training to interpret the visual representation of the skewness analysis.

## Conclusion

Intervals for reliability coefficients can be estimated in various ways. We demonstrated bootstrap and SPSS syntax procedures in this study. There is no particular reliability coefficient or CI estimation method that authors are expected to universally invoke [31,32]. After all, each of the factors in a quality-of-life assessment was different in our study; however, they did not independently vary. We suggest that authors routinely report CIs in quality-of-life publications, to clarify the statistical estimates of the effect of sampling error variance on score reliability. These requirements may also facilitate the understanding that scores in a questionnaire are not completely associated with invariant reliability. Furthermore, using expected response scores is encouraged. Graphical skewness representation with 95% CI is also encouraged to depict patient quality of life in future studies.

## List of abbreviations

CTT: classic test theory; Alpha: Cronbach's *α*; IRT: item response theory; CI: confidence interval; MNSQ: mean square error.

## Competing interests

The authors declare that they have no competing interests.

## Authors' contributions

WC, SJ and LF collected all data, generated the database, designed and performed the statistical analysis and wrote the manuscript. TW, SJ,WW and LF contributed to the development of the study design and advised on the performance of the statistical analysis. The analysis and results were discussed by all authors together. TW, WW and WP contributed to the Excel programming, helped to interpret the results and helped to draft the manuscript. All authors read and approved the final manuscript.

## Supplementary Material

Additional file 1 **Expected scores obtained by the Rasch model's probability theory**. Excel-VBA program for randomly generating Rasch model's expected scores. Click here for file

Additional file 2 **Author-made A Excel-VBA module computing test Cronbach's *α*, item/person skewness coefficient and person expected response score**. Excel-VBA routine of a calculation demonstration for skewness and its plotting. Click here for file

Additional file 3 **SPSS syntax procedures for estimation of the reliability of the 95% CI**. A example of SPSS syntax procedures for Cronbach *α *and its 95% CI. Click here for file

Additional file 4 **The dimensionality and invariance of the EORTC QLQ-C30 explored briefly through the Rasch model**. A brief analysis for the dimensionality of the EORTC QLQ-C30. Click here for file
