# Supplementary material for: Reliability of 95% confidence interval revealed by expected quality-of-life scores: an example of nasopharyngeal carcinoma patients after radiotherapy using EORTC QLQ-C 30
Source: Health Qual Life Outcomes. 2010 Jul 13;8:68. doi: 10.1186/1477-7525-8-68 (PMC2912790; doi:10.1186/1477-7525-8-68)
Supplement: Additional file 1 — Expected scores obtained by the Rasch model's probability theory. Excel-VBA program for randomly generating Rasch model's expected scores. [file 1477-7525-8-68-S1.DOC]

**Additional file 1**: Expected scores obtained by Rasch model’s probability theory

threshold = 3 ‘ this is three step threshold difficulties

item = 28

person = 115

ReDim MEASURE1a(threshold) As Single

For jk = 1 To threshold

MEASURE1a(jk) = Sheets("main").Cells(jk + 2, 3) ‘ loading the values of step difficulties

Next jk

For jkk = 1 To 100 ‘ simulated 100 replication

For j = 2 To person + 1 ‘ for persons

For i = 2 To item + 1 ‘ for items

ability2 = Sheets("poly2").Cells(j, 1) ‘ retrieving the person’s logit ability

all_p = 0

Randomize

mrnd = Rnd

item_diff = Sheets("poly2").Cells(1, i) ‘ the overall item difficulty for each item

If threshold = 3 Then

a0 = 1

a1 = Exp(ability2 - (MEASURE1a(1) + item_diff))

a2 = Exp(2 * ability2 - (MEASURE1a(1) + item_diff) - (MEASURE1a(2) + item_diff))

a3 = Exp(3 * ability2 - (MEASURE1a(1) + item_diff) - (MEASURE1a(2) + item_diff) - (MEASURE1a(3) + item_diff))

all_p = a0 + a1 + a2 + a3

p_a0 = a0 / all_p

p_a1 = a1 / all_p

p_a2 = a2 / all_p

p_a3 = a3 / all_p

all_p1 = p_a0 + p_a1 + p_a2 + p_a3

If mrnd < p_a0 Then

Sheets("poly2").Cells(j, i) = 1

ElseIf mrnd < p_a1 + p_a0 Then

Sheets("poly2").Cells(j, i) = 2

ElseIf mrnd < p_a2 + p_a1 + p_a0 Then

Sheets("poly2").Cells(j, i) = 3

Else

Sheets("poly2").Cells(j, i) = 4

End If

End If

Next i

Next j

Sheets("temp").Cells(jkk + 1, 9) = Sheets("temp").Cells(105, 1) ‘To store the reliability value

Next jkk
