# Supplementary material for: Reliability of 95% confidence interval revealed by expected quality-of-life scores: an example of nasopharyngeal carcinoma patients after radiotherapy using EORTC QLQ-C 30
Source: Health Qual Life Outcomes. 2010 Jul 13;8:68. doi: 10.1186/1477-7525-8-68 (PMC2912790; doi:10.1186/1477-7525-8-68)
Supplement: Additional file 3 — SPSS syntax procedures for estimation of the reliability of the 95% CI. A example of SPSS syntax procedures for Cronbach α and its 95% CI. [file 1477-7525-8-68-S3.DOC]

**Additional file 3**: SPSS syntax procedures for reliability 95% CI estimation

RELIABILITY

/VARIABLES=VAR00001 to VAR00028

/SCALE('ALL VARIABLES') ALL/MODEL=ALPHA.

reliability variables=VAR00001 to VAR00028/

scale(TOTAL)=VAR00001 to VAR00028/

statistics=corr cov/summary=means var total/

icc=model(random) type(consistency) cin=95 testval=.70/

model=alpha.
