# Supplementary material for: Reliability of 95% confidence interval revealed by expected quality-of-life scores: an example of nasopharyngeal carcinoma patients after radiotherapy using EORTC QLQ-C 30
Source: Health Qual Life Outcomes. 2010 Jul 13;8:68. doi: 10.1186/1477-7525-8-68 (PMC2912790; doi:10.1186/1477-7525-8-68)
Supplement: Additional file 4 — The dimensionality and invariance of the EORTC QLQ-C30 explored briefly through the Rasch model. A brief analysis for the dimensionality of the EORTC QLQ-C30. [file 1477-7525-8-68-S4.DOC]

**Additional file 4**

: The dimensionality and invariance of the EORTC QLQ-C30 explored briefly through the Rasch model

*Rasch Analysis of the* EORTC QLQ-C30

***1. Item Fit EORTC QLQ-C30***

The item summary is shown in Table 1 of the manuscript and Figure 1 of this Appendix, in which we can see that the mean and standard deviation of person measures for observed responses(OR) were -2.07 and 1.11) logits, respectively; and they were -2.17 and 1.24 logits, respectively, for expected responses(ER).Using the estimated (or anchored) person, item and structure measures or person-response-string resampling-with-replacement to simulate a data file equivalent to the observed raw data as similar procedures to the previous researches[1-3], we found that the ER yields less mean square errors in a range of 0.5 and 1.5 than the OR shown in manuscript’s Table 1. All of those 28 items demonstrated good fit to the Rasch model.

The item difficulty and person measures were plotted along the same latent trait in order to compare the distribution of item difficulty (location) and person ability (i.e., quality of life) in Figure 1. This allows identification of areas of ability that are poorly assessed by the items [4]. In general, most respondents were located far below items (top X in right-hand item column), suggesting most respondents had a high degree of positive quality of life. This is, the respondents with more than 50% probability to the top category of the hardest item showed a high negative quality of life. The left-hand column of the quality of life locates the person ability measures along the variable. Observe that most of respondents with # symbol are above the bottom category of the easiest item (bottom X in left-hand item column), suggesting few ones have lower 50% probability to the bottom category of the easiest item.

A comparison of the two standard deviations reveals that persons in the measurement of quality of life were dispersed to a wider extent in ER than in OR. The Rasch person separation reliability was 0.89(separation index = 2.80) and 0.86(separation index = 2.51) for ER and OR, respectively. Person measures indicate that these items were easier to endorse on a scale of quality of life from better (coded as 1) to worse (coded as 4) (1 = Not at all, 2 = A little, 3 = Quite a bit, and 4 = Very much).

***2.Item thresholds***

Distances between item thresholds under Rasch rating scale model are shown in the bottom of Table 1. It can be seen that step threshold difficulties are -1.88, 0.73 and 1.15 logits for OR compared to -2.03, 0.59 and 1.34 logits for ER. According to the guidelines for the appropriateness of level of scaling categorization, there were eight guidelines proposed by Linacre [5]. Some of them regarding Item thresholds are shown as followings: (3) average measures advance monotonically with category, (7) step difficulties advance by at least 1.4 logits, and (8) step difficulties advance by less than 5.0 logits. We can see from those guidelines that only gap of step difficulties exhibits slightly small in the last two thresholds. The lack of threshold disordering supports the use of the Likert scoring method [6] for the EORTC QLQ-C30.

***3. Dimensionality of EORTC QLQ-C30***

Unidimensionality can be assessed based on a principal component analysis (PCA) of residuals[6,7]. Smith[7] suggested using an independent t-test approach to compare estimates of person locations based on different item subsets[8,9]. If deviation from unidimensionality is trivial, the number of person locations that differ between the two item sets is small. Wright[10] proposed that R(unidimension: the ratio of Rasch real person reliability to the model one) above 0.9 indicates a clearly unidimensional variable, value below 0.5 might be cause for alarm[10].

The PCA decomposes the item correlation matrix based on standardized residuals, or differences between what is observed and what is predicted, [11] to determine whether there are other potential dimensions. The first factor of the PCA is the Rasch dimension. A variance of 60% or greater accounted for by the Rasch dimension is considered to be good [11]. The second dimension, or the first contrast in the residuals, can suggest whether there are any patterns in the differences within the residuals large enough to suggest that more than one dimension exists. If there is little variance accounted for by the Rasch dimension, but significant amounts of variance within the contrast dimensions, the scale may be multidimensional. A rule of thumb when working with residual-based PCA is that the secondary dimension should have the strength of at least three items (as measured by its eigenvalue) to be considered a possible second dimension and should represent more than 5% of the unexplained variance [11].

The scree plot (in Figure 2) of the variance components and sizes of percent variance logarithmically scale was plotted for ER, in which the distance vertically from T to TV on X-axis means total variance in the observations, always 100%; M to MV means variance in the observations explained by the Rasch measures; U to UV was unexplained variance; P and I indicate raw variance explained by persons and item, respectively; 1 to U1: first contrast (component) in the residuals; 2 to U2: second contrast (component) in the residuals, etc. When existed multidimensionality for a scale, the first contrast (also called first residual factor of which the magnitude against the Rasch factor) simplified as 1 in the scree plot will be visually significantly apart from the second contrast as 2.

Figure 2 of this Appendix displays the PCA results and loadings (i.e., the relation of the item to the underlying single construct) for the QLQ-C30 scale response model.

The principal components analysis of the residuals demonstrated that a 28-item scale (EORTC QLQ-C30) accounted for 38.7% of the empirical variance for ER and 36.5% for OR. Both of which below the desired 60% are explained by the model. The model’s variance of 39.4% is almost the same as the empirical one for ER, indicating the data set fit the Rasch model well. The first contrast had an eigenvalue of 2.3 (less than 3) and accounted for 5.0% and 6.9% for ER and OR, respectively (equal to 5% of the unexplained variance) of the unmodeled data, suggesting that the QLQ-C30 scale for ER is unidimensionality.

***4. Differential Item Functioning of EORTC QLQ-C30***

DIF analysis was conducted to assess invariance of item-difficulty hierarchy across groups of respondents (e.g., cancer stage in this study). An item is deemed to display DIF if the response probabilities for that item cannot be fully explained by the latent trait. DIF analysis identifies items that appear to be unexpectedly too difficult or too easy for a particular group of respondents. The test construct must remain invariant across groups of cancer stage. DIF analysis is a way of verifying construct equivalence over groups. All items ought to be DIF-free or at least DIF-trivial in order to obtain comparable measures among the groups [12]. A difference larger than 0.5 logits (equal to an odds ratio of 1.65) in the difficulty estimates between any groups was treated as a substantial DIF [6,12-14]. No differential item functioning was exhibited in group of cancer stage for any item of the EORTC QLQ-C30 by OR or ER.

**References:**

1. Harwell M., Stone CA, Hsu TC, Kirisci L: **Monte Carlo studies in item response theory**. *Applied Psychological Measurement 1996*, **20**, 101-125.
2. Macdonald P, Paunonen SV:**A monte carlo comparison of item and person statistics based on item response theory versus classical test theory**. *Educational and Psychological Measurement 2002*, **62**: 921-943.
3. Chien TW, Wu HM, Wang WC, Castillo RV, Chou W: **Reduction in patient burdens with graphical computerized adaptive testing on the ADL scale: tool development and simulation**. *Health Qual Life Outcomes. 2009*; 7:39.
4. Smith AB, Wright EP, Rush R, Stark DP, Velikova G, Selby PJ. **Rasch analysis of the dimensional structure of the Hospital Anxiety and Depression Scale**. *Psychooncology* 2006; **15**(9):817-27.
5. Linacre JM: **Optimizing Rating Scale Category Effectiveness**. *Journal of Applied Measurement 2002*;3(1): 85-106.
6. Smith AB, Fallowfield LJ, Stark DP, Velikova G, Jenkins V: **A Rasch and confirmatory factor analysis of the General Health Questionnaire (GHQ)-12**. *Health and Quality of Life Outcomes 2010*, **8**: 45.
7. Smith EV. Jr. **Understanding Rasch measurement: Detecting and evaluating the impact of multidimensionality using item fit statistics and principal component analysis of residuals**. J *Applied Measurement 2002,* ***3****(2)*, 205-231.
8. Tennant A, Pallant J: **Unidimensionality matters**. *Rasch Meas Trans 2006*, **20**:1048-1051. [

](http://www.hqlo.com/sfx_links.asp?ui=1477-7525-6-47&bibl=B38)
9. Wann-Hansson1 C, Klevsgård R, Hagell P. **Cross-diagnostic validity of the Nottingham health profile index of distress (NHPD)**.*Health and Quality of Life Outcomes 2008*, **6**:47.
10. Wright BD.**Unidimensionality coefficient**. *Rasch Measurement Transactions, 1994*, 8:3, 385
11. .LinacreJM. **WINSTEPS [computer program]**. Chicago, IL: http://www.WINSTEPS.com. 2008.
12. Shih CL,Wang WC: **Differential item functioning detection using the multiple indicators, multiple causes method with a pure short anchor**. *Applied Psychological Measurement* 2009; **33**: 184-199.
13. Holland PW, Wainer H: *Differential Item Functioning*. Hillsdale, NJ: Erlbaum, 1993.
14. Lai JS, Cella D, Chang CH, Bode RK, Heinemann AW: **Item banking to improve, shorten and computerize self-reported fatigue: an illustration of steps to create a core item bank from the FACIT-Fatigue Scale**. *Qual Life Res 2003*, 12: 485-501.

MEASURE | BOTTOM P=50% | MEASURE | TOP P=50% MEASURE

<more> ---- Patient -+- Item -+- Item -+- Item <rare>

4 + + + 4

| | | X

| | |

| | |

| | |

| | |

3 + + + X 3

| | |

| | | X

| | | X

| | X |

| | | XXXX

2 + + + 2

| | | XX

X | | | X

| | X |

| | | XXXXXX

| | X | XX

1 + + X + 1

| | | XXX

X | | XXXX | XXX

| | | XXX

| X | XX |

X | | X |

0 X + + + 0

| | XXXXXX |

| | XX |

XXX | X | |

XX | | XXX |

XXXX | X | XXX |

-1 XXXX + X + XXX + -1

XXXXX | | |

XXXX | XXXX | |

XXXXXXXXX | | |

XXXXXXXXXX | XX | |

XXXXXXXXXX | X | |

-2 XXXXXXXXXXXX + + + -2

XXX | XXXXXX | |

XXXX | XX | |

XXXXX | | |

XXXXXXX | XXX | |

XXXX | XXX | |

-3 XXXXXX + XXX + + -3

XXX | | |

| | |

XXXX | | |

XXX | | |

XXX | | |

-4 + + + -4

X | | |

| | |

| | |

X | | |

| | |

-5 + + + -5

| | |

| | |

XX | | |

| | |

| | |

-6 XX + + + -6

<less> ----- Patient-+- Item -+- Item -+- Item <frequ>

Figure 1 Map of persons (left) and items (right) for observed responses

Note. Rasch person separation reliability is 0.86 and 0.89 for observed and expected responses, respectively.

Table of STANDARDIZED RESIDUAL variance (in Eigenvalue units)

-- Empirical -- Modeled

Total raw variance in observations = 45.7 100.0% 100.0%

Raw variance explained by measures = 17.7 38.7% 39.4%

Raw variance explained by persons = 8.2 17.9% 18.3%

Raw Variance explained by items = 9.5 20.7% 21.1%

Raw unexplained variance (total) = 28.0 61.3% 100.0% 60.6%

Unexplned variance in 1st contrast = 2.3 5.0% 8.1%

Unexplned variance in 2nd contrast = 2.0 4.4% 7.2%

Unexplned variance in 3rd contrast = 2.0 4.3% 7.0%

Unexplned variance in 4th contrast = 1.7 3.6% 5.9%

Unexplned variance in 5th contrast = 1.6 3.6% 5.8%

STANDARDIZED RESIDUAL VARIANCE SCREE PLOT

VARIANCE COMPONENT SCREE PLOT

+--+--+--+--+--+--+--+--+--+--+--+

100%+ T +

| |

V 63%+ +

A | U |

R 40%+ +

I | M |

A 25%+ +

N | I |

C 16%+ P +

E | |

10%+ +

L | |

O 6%+ +

G | |

| 4%+ 1 2 3 +

S | 4 5 |

C 3%+ +

A | |

L 2%+ +

E | |

D 1%+ +

| |

0.5%+ +

+--+--+--+--+--+--+--+--+--+--+--+

TV MV PV IV UV U1 U2 U3 U4 U5

VARIANCE COMPONENTS

Figure 2 STANDARDIZED RESIDUAL VARIANCE SCREE PLOT for QLQ-H&N35 of expected responses
